# Virus taxonomy and the ICTV – 21 FAQs for the perplexed virologist

**DOI:** 10.1099/jgv.0.002243

**Published:** 2026-05-13

**Authors:** Donald B. Smith, Peter Simmonds, Stuart G. Siddell

**Affiliations:** 1 Nuffield Department of Medicine, University of Oxford, Oxford, UK; 2 Virology, Institute of Biomedicine, University of Turku, Turku, Finland; 3 School of Cellular and Molecular Medicine, University of Bristol, Bristol, UK

**Keywords:** International Committee on Taxonomy of Viruses (ICTV), virus taxonomy

## Abstract

Just over 125 years has passed since the ‘filterable’ agents of tobacco mosaic disease and foot-and-mouth disease were first described as infectious, replicating entities smaller than bacteria. Today, viruses are formally classified into more than 16,000 species ranked into genera, families and higher taxa. The development of an official virus taxonomy has been overseen by an International Committee, first constituted in 1966 and renamed as the International Committee on Taxonomy of Viruses (ICTV) in 1975. Despite the engagement of the ICTV in virus taxonomy over the last 60 years, many aspects of virus classification and nomenclature may seem odd or sometimes incomprehensible to virologists more familiar with the taxonomy of cellular organisms. Who runs the ICTV? What are virus species demarcation criteria? Why have all virus species names become binomial? How can a sequence in a metagenomic dataset be assigned to a virus species? This article attempts to answer several such questions and outlines how a large, inclusive and global community of virologists has developed new and responsive policies for virus taxonomy in a decade when the pace of virus discovery has dramatically accelerated.

## Introduction

An article written to commemorate the International Committee on Taxonomy of Viruses (ICTV)’s 50th anniversary [1] commented that ‘Virus taxonomy, like any biological taxonomy, is never perfect or complete’. The truth of this statement is supported by developments overseen by the ICTV over the last decade, including important changes to the nomenclature of species, the structure and scope of taxonomic ranks and the increasing use of genomics-based classification for taxonomic assignments [2,4]. Change is always difficult to navigate and this may tempt some to dismiss virus taxonomy as complex, peripheral or even unreliable.

However, many of the adjustments recently introduced by the ICTV were designed to stabilize and strengthen virus taxonomy and to help structure our understanding of the evolutionary and biological relationships between different viruses. These changes reflect a new awareness of the extent and complexity of the virosphere, encompassing a range of genetic diversity paralleling that of eukaryotes and prokaryotes.

Within the last decade, the number of virus taxa has increased fivefold, including new assignments at higher taxon levels such as realm, kingdom and phylum. as well as the lower levels of family, genus and species. Viruses are increasingly found in association with a wider variety of hosts, including other viruses, and they infect organisms that inhabit some of the world’s most extreme habitats. This burgeoning of virus taxonomy has been driven by the application of high-throughput sequencing and a range of innovative bioinformatic tools developed to analyse a large volume of metagenomic data. To keep pace, the ICTV has been working to develop analytical tools that support the creation, expansion, and management of virus taxonomy and to provide a stable, responsive and scalable information technology platform.

Virus taxonomy is the product of the work of many virologists with an interest in taxonomy, or who have contributed knowledge about virus genome structure and biology. Now is an ideal time for virologists to learn more about what the ICTV has accomplished over the last few decades. Accordingly, this article answers a series of key questions about virus taxonomy (Box 1) that a curious virologist might ask.

Box 1. **Links to questions that a curious virologist might ask**

Carl Linnaeus, later known as Carl von Linné, the founder of the systematic classification of animals and plants, could not have anticipated how these principles would be applied in modified form to viruses. Portrait by Alexander Roslin, Swedish National Museum.

1. Who invented virus taxonomy?

2. Just who is in charge of virus taxonomy?

3. Is the ICTV a sinister cabal?

4. How can I get involved in virus taxonomy?

5. Does the ICTV only deal with viruses?

6. Why do virus names keep changing?

7. So, who regulates virus names?

8. What is a biological virus species?

9. What are demarcation criteria?

10. Am I a lumper or a splitter?

11. Why does the ICTV keep updating virus species names?

12. Why have all virus species names become binomial?

13. Why does the ICTV use a 15-rank taxonomic framework?

14. How can a virus family become an order?

15. What are the special features of virus taxonomic names?

16. How do I know if a virus I have discovered belongs to a new species?

17. How can you classify viruses that have not been isolated?

18. Why are there millions of virus genome sequences but only thousands of virus species?

19. How can I find out more about virus taxonomy?

20. What does the future hold for virus taxonomy?

21. What would we like ChatGPT to say about virus taxonomy and the ICTV?

**1. Who invented virus taxonomy?**

In the first half of the 20^th^ century, several attempts were made to create a framework for virus taxonomy [5]. However, there was uncertainty about whether an evolutionary-based classification of viruses was possible [6], and there was intense debate about what features of viruses should be used in classification [7]. When formal responsibility for virus taxonomy was given to the International Committee on Nomenclature of Viruses (ICNV) in 1966 (renamed as the ICTV in 1975), these debates continued. Nevertheless, in the first 50 years of the ICTV, the taxonomy of viruses developed from placing a few hundred viruses into genera and families [8] to the acceptance of the species concept for viruses [9], the use of a five-tier hierarchical ranking (species, genus, subfamily, family, order) [10] and the classification of about 4,500 virus species [11] (Table 1). Unfortunately, this period was also marked by both conceptual and practical changes to virus taxonomy that were a source of some confusion. However, many of these issues are largely settled, and virus taxonomy is now organized in a similar way to the taxonomies of other organisms.

**Table 1.** Landmarks of ICTV taxonomy

1966	Establishment of the International Committee on Nomenclature of Viruses (ICNV)
1971	First classification of viruses into groups, genera and families
1971	Rejection of the law of priority for naming virus taxa
1971	Publication of the first ICTV Report (81 pages)
1975	Renaming of the ICNV to the ICTV
1991	Recognition of the species concept for viruses (and designation of representative isolates)
1991	Introduction of a 5-tier hierarchical ranking (species, genus, subfamily, family, order)
1991	Publication of the first Virus Division News (VDN) article in Archives of Virology
1995	Creation of the ICTV website
2011	Publication of the ninth and last printed edition of the ICTV Report (1,327 pages)
2017	Online-only publication of the ICTV Report (10^th^ Edition) and associated Profiles published in the Journal of General Virology
2017	Classification of viruses identified solely by metagenomics or metatranscriptomics
2020	Introduction of a 15-tier hierarchical ranking
2021	Introduction of mandated binomial format for virus species names
2023	Adoption of an evolutionary relationship-based virus classification

**2. Just who is in charge of virus taxonomy?**

The ICTV is an entity under the Virology Division of the International Union of Microbiological Societies (IUMS) made up of voluntary members (see Q3. Is the ICTV a sinister cabal?), which is charged with developing, refining and maintaining a universal virus taxonomy. This taxonomy informs public resources such as NCBI Taxonomy. The ICTV has sole responsibility for virus taxonomy. A consequence of this is that, unlike other branches of biology, the taxonomy of viruses only changes through decisions made by the membership of the ICTV. The establishment of a new virus species (or other taxon), or a change to the existing taxonomy, requires that a Taxonomic Proposal is submitted to the ICTV and the proposed taxonomic changes only come into effect once the proposal has been scrutinized for accuracy and consistency by the ICTV Executive Committee (EC) [and any appropriate ICTV Study Groups (SGs)] and then ratified by a vote of the entire ICTV membership. The process, from submission of a proposal to ICTV ratification, usually takes 9–12 months.

**3. Is the ICTV a sinister cabal?**

**Fig. 1.**
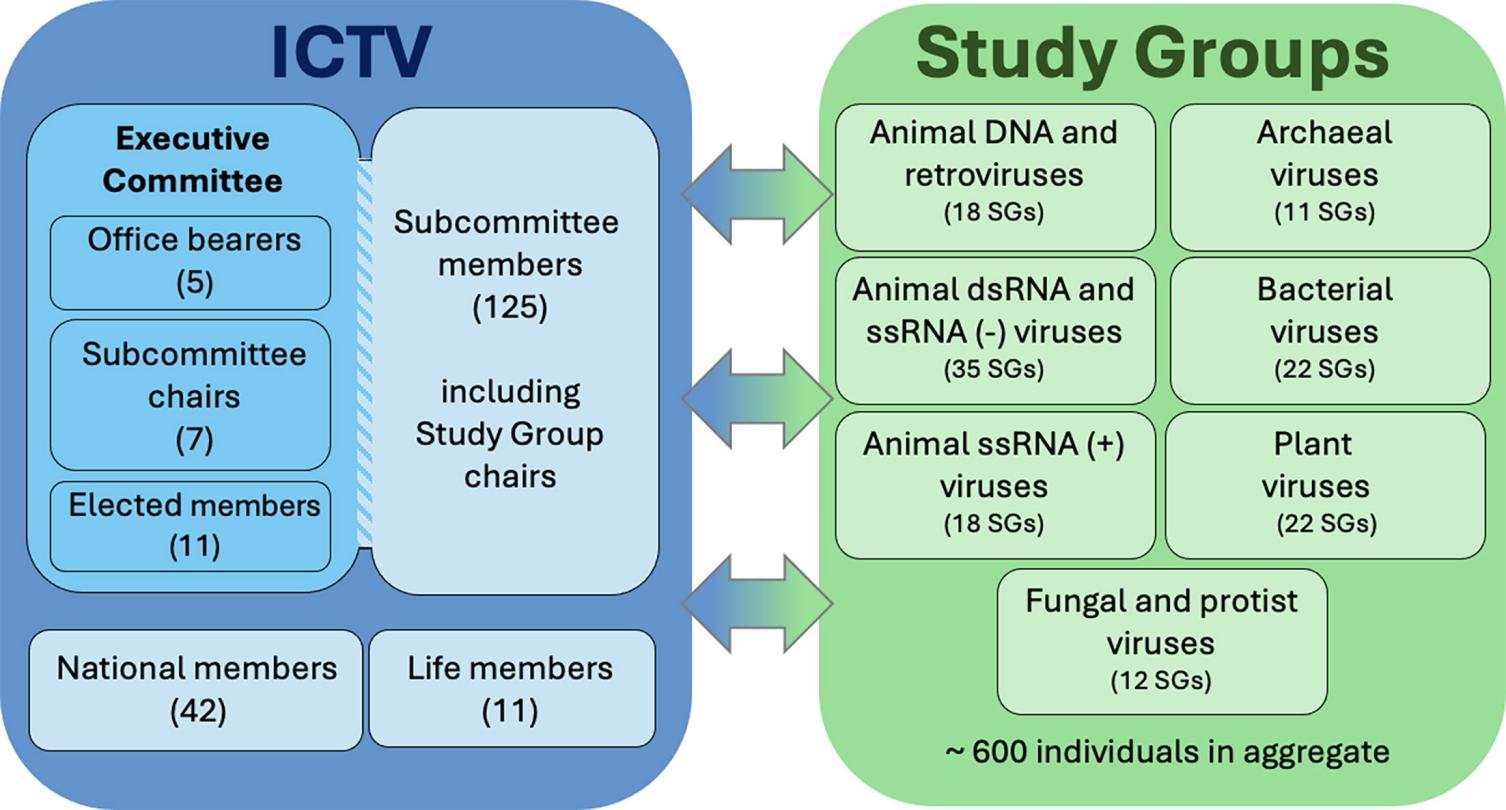
Organization of the ICTV and SGs in 2026. Voting members of the ICTV (blue box) include 23 EC members, 42 national members, 11 life members and subcommittee members of whom 84 are additional to the other groups. The 138 subcommittee-affiliated SGs established by the ICTV (green box), and the recently formed Bioinformatics SG, include about 600 individuals in aggregate, some of whom are also members of the ICTV.

No! The ICTV is neither sinister nor a cabal but is an outward-facing, community-engaged and democratically elected group of virologists who manage the policy and practice of virus taxonomy. There are several elements to the membership of the ICTV, as well as hundreds of individuals in SGs who contribute to its work (Fig. 1) [12]:

- The ICTV EC is comprised of 5 office bearers, 7 Subcommittee Chairs (each responsible for a subsection of viruses, for example, archaeal viruses, plant viruses, animal ssRNA(+) viruses, etc.) and 11 elected members. These EC members are elected by the full membership of the ICTV every 3 years.
- National members of the ICTV (up to two representatives from each country, as nominated by a virological society from that country).
- Life members of the ICTV (appointed by the EC for their previous contribution to virus taxonomy).
- SG chairs appointed to further the taxonomy of particular virus families or other taxa.
- SG members drawn from the wider virology community who advise on taxonomy and co-author ICTV Report chapters.

**4. How can I get involved in virus taxonomy?**

Anyone can participate in developing virus taxonomy by submitting a Taxonomic Proposal to the ICTV, consisting of a Word document and an Excel document. Templates and instructions for these documents can be found on the ICTV . Proposals are initially evaluated for conformity to ICTV rules by Subcommittee Chairs, reviewed in detail by the EC at its annual meeting, and if accepted by the EC, they are considered for ratification by a vote of the full ICTV membership. Ratified taxonomic additions or changes appear in the updated Master Species List (MSL) and associated outputs (see Q19. How can I find out more about virus taxonomy?). Authors of all ratified proposals automatically become authors of a MEDLINE-indexed publication that summarizes changes in taxonomy for each of the seven ICTV subcommittees [13].

Individuals can also contribute to the work of the ICTV as a member of an ICTV SG. Membership of SGs is managed by each SG chair who is appointed by the appropriate Subcommittee Chair. Also, virus taxonomy has little value unless it is used. Everyone can support the ICTV by using virus taxonomic names appropriately when writing articles. For example, when a virus is first mentioned, it is helpful to name the virus species that it has been assigned to, and perhaps also the family or other higher ranks.

**5. Does the ICTV only deal with viruses?**

The ICTV Code describes a virus as a mobile genetic element that encodes at least one protein that is a major component of the virion. While viruses, so defined, constitute the vast bulk of the ICTV taxonomy, the classification extends to other virus-like entities that replicate in cellular organisms. The current taxonomy includes viruses that are dependent on other viruses for packaging, an example being hepatitis D virus (family *Kolmioviridae*). Similarly, the ICTV classifies viruses that no longer form infectious particles, for example, fungal viruses in the family *Hypoviridae* that lack an extracellular phase, self-replicating RNAs such as viroids that do not encode any proteins, viriforms (mobile genetic elements that have become incorporated into their host genome and produce non-infectious virions) and polydnaviriforms.

**6. Why do virus names keep changing?**

They don’t! Most virus names have been remarkably stable over the many decades of virus discovery. Changes in virus names are the exception rather than the rule and typically are the result of the virus community recognizing a lack of clarity or inappropriateness of an initially coined name. For example, hepatitis G virus is now referred to as human pegivirus type 1 because it is no longer thought to cause hepatitis.

The misconception that virus names often change stems, in part, from confusion about the distinction between virus names and virus species names [4] (see Q11. Why does the ICTV keep updating virus species names?). For example, the agent that was discovered to be the principal cause of non-A, non-B hepatitis was named hepatitis C virus in the initial publication [14] and that is still the name of the virus today. However, the name of the species to which this virus is assigned has changed several times – initially the species was named *Hepatitis virus C* (in 1991), then *Hepatitis C virus* (in 1995), *Hepacivirus C* (in 2017), *Hepacivirus hominis* (in 2023) and from 2026 *Orthohepacivirus hominis* [15]. However, these kinds of changes to virus species names have no impact on virus names.

**7. So, who regulates virus names?**

Officially, no-one! Virus names are chosen by the virologists who study them, often the person who first described the virus. There is no such thing as an official virus name, and the same virus may have different names in different languages; foot-and-mouth disease virus, Maul-und-Klauenseuche-virus and virus de la fièvre aphteuse are all names for the same virus. A virus can also have multiple names in the same language, an example being human gammaherpesvirus 4 and Epstein–Barr virus. Although the ICTV has no statutory remit for virus names, it encourages their consistent usage through its curation of the Virus Metadata Resource (VMR), a spreadsheet that associates each virus species with the English name(s) of one or more named viruses assigned to it. The ICTV also encourages the use of virus names without italics or initial capital letters (for example, measles virus) to avoid confusion with virus species names (in this case, *Morbillivirus hominis*). Guidance on how to write virus species names and advice on the use of virus names is available on the ICTV website and in Zerbini *et al.* [16].

**8. What is a biological virus species?**

According to the ICTV Code:

*‘A biological species is a monophyletic group of viruses whose properties can be distinguished from the properties of other species by multiple criteria*.’

This definition means, in essence, that viruses in a biological species form a genetically coherent group with a shared evolutionary origin and that members match one or more defining criteria. These demarcation criteria usually involve measures of genetic similarity but can also extend to phenotypic properties such as disease associations, host range and geographical range (see Q9. What are demarcation criteria?).

The variability in criteria used for species definitions contributes to the considerable uncertainty about the nature of virus species, and the extent to which artificial partitioning of virus diversity by taxonomists equates to real or useful divisions of viruses. However, debate on this issue need not derail the entire enterprise of virus taxonomy! The current definition of virus species allows many aspects of virus biology and ecology to be flexibly used as demarcation criteria and generally facilitates a meaningful and practical classification of viruses at the lowest taxonomic rank.

**9. What are demarcation criteria?**

A proposal for a new species, or for changes to the classification of existing species, requires that demarcation criteria are defined that can be used to decide whether or not a particular virus belongs to that species. These criteria have previously included both biological and physiochemical properties. More recently, demarcation criteria have tended to be framed around genetic relatedness. Most virus species have multiple demarcation criteria, and it is preferable to use as many as possible. The only requirement is that they are all congruent and that members of the species so defined share a common evolutionary origin [3].

Demarcation criteria are, in principle, required for all taxa, but at higher ranks, they may be very broad and focus on a single hallmark gene involved in virus replication, virion structure or virion morphogenesis. As analytical approaches continue to evolve and our understanding of evolutionary relationships deepens, demarcation criteria are likewise being refined to include features such as the secondary structure of proteins.

**10. Am I a lumper or a splitter?**

Some virologists (lumpers) think of a virus species as a population of viruses with a broad range of biological and physical attributes that are, nevertheless, capable of exchanging genetic information. For example, all isolates of influenza A virus, whatever their host or haemagglutinin or neuraminidase composition (with the possible exception of the bat influenza A virus strains), are considered as belonging to the species *Alphainfluenzavirus influenzae*, the sole species in its genus. The rationale for this is that since reassortment of virus genome segments can occur between any of these viruses, they belong to a single ‘biological’ entity.

In contrast, other virologists (splitters) choose to distinguish viruses on the basis of particular biological and ecological properties. Thus, the flaviviruses West Nile virus (species *Orthoflavivirus nilense*) and Usutu virus (species *Orthoflavivirus usutuense*) are assigned to separate species due to their distinct geographical ranges and pathogenicity. This is despite the viruses in these species having less genetic divergence from each other than exists between the four serotypes of dengue virus, which are all assigned to one species, *Orthoflavivirus denguei*.

**11. Why does the ICTV keep updating virus species names?**

There are examples of virus species names that have changed multiple times in the six decades since the ICTV was established (see Q6. Why do virus names keep changing?) [5]. However, changes to taxonomic names are not confined to virology – bacterial species names and their underlying taxonomy have been punctuated by extensive reorganization at both species and genus rank, as exemplified in the genus *Pseudomonas* [17]. Expectations of stability may arise because we are generally more familiar with the taxonomy of plants and animals that has developed over 250 years. Many animal and plant species were assigned and named in the 18th century and have not since needed to be revised. The relatively short history of virus taxonomy, together with the rapid expansion in our knowledge of the virosphere in recent years, may be giving the false impression that this fluidity is frivolous.

**12. Why have all virus species names become binomial?**

The most visible and controversial recent change to virus taxonomy has been the mandated use of binomial names for virus species [4]. Previously, virus species names had been formulated in a variety of formats that were mostly inconsistent with the Latinized binomial name format adopted in the rest of biology [18]. To address this anomaly, the ICTV decided in 2021 that from 2024 onwards, all virus species names would follow a format similar to the Latinized binomials used for organisms, the requirement being that virus species names consist of two words, first the genus name and then a species epithet. While Latinization is permitted, species epithets can be freeform, although restricted to letters of the Latin alphabet and numbers without punctuation. The major advantages of the binomial rule are that virus species have become recognizable and distinct from the names of viruses assigned to those species and that the genus assignment of a virus species is obvious from its species name.

**13. Why does the ICTV use a 15-rank taxonomic framework?**

**Fig. 2.**
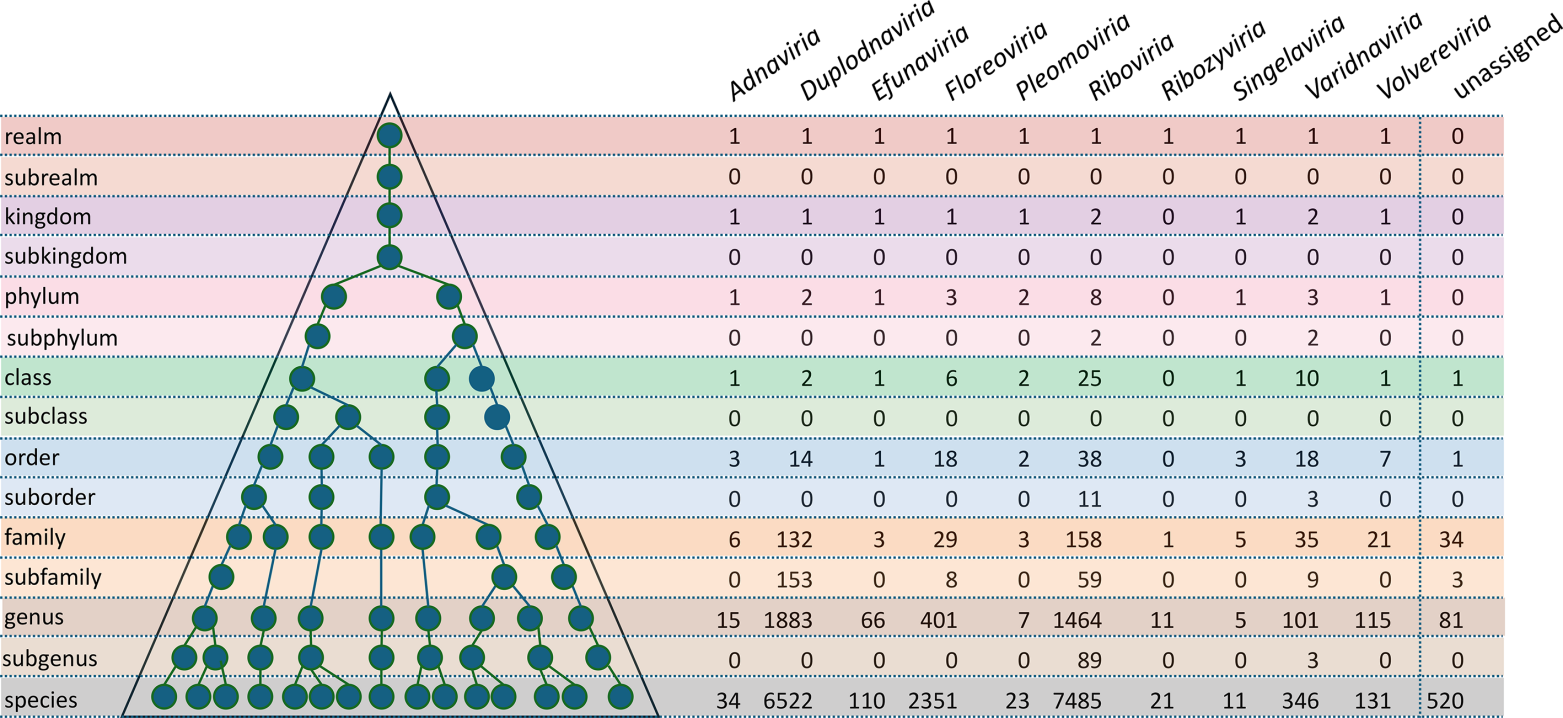
The ICTV taxonomic framework. The current ICTV virus taxonomy is a 15-tier ranked system that can be depicted as a pyramid representing the phylogeny of virus lineages. Each circle in the hypothetical lineage represents a virus taxon. The numbers of taxa are given for each rank within each of the ten realms; 640 taxa are not assigned to a realm. The subrealm, subkingdom and subclass ranks are currently unused.

Hierarchical virus classification organizes viruses into a series of groups (i.e. taxa) within groups, from the broadest (highest rank) to the most specific (lowest rank). This results in a classification system that, with the appropriate nomenclature, facilitates communication and helps to identify similarities and differences between taxa, ideally including their evolutionary relationships. In 2020, the ICTV introduced a series of higher ranks (realm, kingdom, phylum and class), as well as intermediary ranks such as subrealm and subkingdom (Fig. 2). By adding these ranks to the existing ranks of order, family, subfamily, genus and species, the taxonomy was better able to capture groupings between distantly related viruses that were being revealed by the discovery of new viruses and the application of deeper evolutionary-based genomic comparisons and protein structure analyses [2].

In virus taxonomy, the highest rank of realm is used in place of domain, the highest rank in the Linnaean classification scheme for cellular organisms. The name realm was chosen to reflect the evidence that, unlike the last universal common ancestor, from which all cellular life is presumed to have descended, viruses have arisen independently on multiple occasions [19,21].

**14. How can a virus family become an order?**

Ideally, taxa at the same rank would represent viruses with similar degrees of genetic diversity. That is why, as genomic analyses of virus lineages become more extensive, taxa may be promoted or demoted between ranks. For example, in 2024, the order *Bunyavirale*s was promoted to the class *Bunyaviricetes* to accommodate a rapidly increasing number of related arthropod and rodent viruses with segmented, negative-sense RNA genomes [22]. Similar considerations led to the re-classification of flavivirus genera into three families, *Flaviviridae*, *Pestiviridae* and *Hepaciviridae* [15]. Further such changes can be expected as genomic and protein structure comparison methods are increasingly used to uncover deeper evolutionary relationships between viruses and to guide taxonomic assignments.

**15. What are the special features of virus taxonomic names?**

In general, the system for nomenclature of virus taxa resembles those used for other organisms. However, a decision taken at the founding of the ICVN was that virus species name would not be based on published authority, unlike the taxonomy of cellular organisms, where valid publication of a description and name of a species constitutes its official taxonomic record and priority. In terms of formatting, virus taxonomic names at all ranks are written using italics in common with the recommended practice for other biological codes (with the notable exception of the *International Code of Zoological Nomenclature*) [23]. Therefore, one would write ‘The plant pathogen cowpea mosaic virus belongs to the species *Comovirus vignae*, which is placed in the family *Secoviridae*, order *Picornavirales*, class *Pisoniviricetes*, phylum *Pisuviricota*, kingdom *Orthornavirae* and realm *Riboviria*’. Virus taxonomic names have codified suffixes associated with particular ranks, these being *-virus* for genus names and *-viridae* for family names (Table 2), with similar suffixes for satellites, viroids and viriforms.

**Table 2.** Taxon rank suffixes for viruses

Rank	Taxon	
	Suffix	Example
Realm	*…viria*	*Riboviria*
Subrealm	*…vira*	
Kingdom	.*…virae*	*Orthornavirae*
Subkingdom	*…virites*	
Phylum	*…viricota*	*Duplornaviricota*
Subphylum	*…viricotina*	
Class	*…viricetes*	*Resentoviricetes*
Subclass	*…viricetidae*	
Order	*…virales*	*Reovirales*
Suborder	*…virineae*	
Family	*…viridae*	*Sedoreoviridae*
Subfamily	*…virinae*	
Genus	*…virus*	*Rotavirus*
Subgenus	*…virus*	
Species	No specific suffix; can be Latinized	*Rotavirus alphagastroenteritidis*

**16. How do I know if a virus I have discovered belongs to a new species?**

The first step is to characterize the virus, with the minimal requirement that a complete annotated genome sequence is obtained and made available on an International Nucleotide Sequence Database Collaboration (INSDC) database. Comparison of the virus genome with those of classified viruses using sequence comparison resources [24] and references in [3] or the ICTV tool TaxaBLAST to identify its closest relatives. Having perhaps identified a family or genus that includes similar viruses, the next step would be to check the demarcation criteria currently in place for those taxa. This information can often be found within the relevant ICTV Report chapters, or if a Report is not available, from examination of the Taxonomic Proposals establishing or revising those taxa. Finally, if there is good evidence that the virus under investigation belongs to a new species, a Taxonomic Proposal should be submitted to the ICTV (see Q4. How can I get involved in virus taxonomy?).

**17. How can you classify viruses that have not been isolated?**

The application of high-throughput sequencing methods to environmental samples has been a particularly fertile source of novel viral sequences. Following an ICTV-organized workshop in Boston, USA, in 2017, the ICTV accepted the principle that viruses identified in metagenomic studies could be incorporated into the taxonomic framework [25]. Subsequently, a large group of virologists (including many ICTV EC members) proposed a minimum reporting standard when depositing the sequence of an uncultivated virus onto a public database [26]. This standard includes descriptions of sample origin, metrics of genome quality and completeness, gene annotations, estimates of biogeographic distribution and *in silico* host prediction. This proposal has now been formalized into specific recommendations by the ICTV [27]. Thus, the coding-complete virus genome sequence has become a primary resource for virus taxonomy purposes and ensures that the assignment of viruses at all ranks within a taxonomy is consistent with inferences about their evolutionary relationships [3]. This new policy has led to a dramatic increase in the number of virus species assigned by the ICTV from 4,853 in 2018 to the current total of 17,554.

**18. Why are there millions of virus genome sequences but only thousands of virus species?**

At present, there are more than 14 million virus sequences listed on the NCBI virus website but just over 17,500 virus species. One reason for this discrepancy is that there are often multiple sequences of different virus isolates on the NCBI Virus database, all belonging to the same species. The best example is SARS-CoV-2, for which there are over nine million NCBI virus entries, all of which are assigned to the single species *Betacoronavirus pandemicum*. Another factor is that many virus sequences are incomplete, and the ICTV requires at least a complete-coding sequence of a virus genome before classification can be considered. And as a matter of procedure, virus taxonomy will always lag behind virus discovery, because the establishment of a virus species by the ICTV requires the submission, approval and ratification of a Taxonomic Proposal in order to establish a new species. The development of automated methods for taxonomic assignments may improve this processing time [24,28, 29].

**19. How can I find out more about virus taxonomy?**

The ICTV provides definitive information about virus taxonomic relationships and names through several tools and resources on its website, https://ictv.global. The current taxonomy can be downloaded as an Excel spreadsheet (the MSL) or can be searched using the Taxonomy Browser tool or explored using the Visual Taxonomy Browser tool. Results obtained using these tools include the history of each taxon, including previous names, changes to its lineage and links to the Taxonomy Proposals that initiated taxonomic changes. The etymology of taxon names is provided for taxa at the rank of family and above (Etymology of Taxon Names). More detailed information about the biology and structure of viruses in particular families can be obtained from individual chapters of the ICTV Report. Accompanying these Report chapters are short ‘Profiles’ published in the Journal of General Virology, acting as the citation for each full Report chapter. To identify viruses that have been assigned to a taxon, the ICTV provides a downloadable Excel spreadsheet called the VMR. This provides, for each virus species, the name of one or more viruses that belong to the species, along with genome accession numbers and general information such as the type of genomic nucleic acid and host. The other side of the coin – associating a particular virus name, isolate name, genome accession number or disease name with a virus species – can be explored using the Find the Species tool.

**20. What does the future hold for virus taxonomy?**

With the introduction of higher ranks and mandated binomial names, it is hoped that virus taxonomy has now entered a period of relative structural and nomenclatural stability. However, this does not mean that virus taxonomy will stagnate – there is likely to be ongoing, rapid expansion in the numbers of virus species, genera, families and higher taxa. The rate at which new virus species are added may accelerate if it becomes possible to automate aspects of the Taxonomic Proposal process, a topic currently being considered by the newly formed ICTV Bioinformatics SG.

Substantial changes can also be expected in the organization of viruses within higher taxonomic ranks as the methods for comparing viruses that are distantly related are developed. An example of this is the recent splitting of the realm *Varidnaviria* [30] to take account of evidence for two independent origins of the major capsid proteins based upon a comparison of their structural folds [20]. Further development of the higher rank taxonomy of viruses is likely as new analytical methods are introduced. For example, increasing use is being made of protein structure comparisons deriving from computational predictions based upon protein sequences [31].

At the other end of the scale, an omission from the current remit of the ICTV concerns classification below the species level. This is important because virus strains may differ in pathogenicity within a host. For example, in humans, the enteroviruses poliovirus 1, poliovirus 2 and poliovirus 3 are neuropathogenic but coxsackievirus A1 and enterovirus C95 are not. Yet all of these viruses are placed in the species *Enterovirus coxsackiepol* and share a common taxonomic description. These differences in the biology of sub-specific variants can have important regulatory or clinical implications, and this is an issue that will have to be resolved by further discussion by the ICTV.

**21. What would we like ChatGPT to say about virus taxonomy and the ICTV?**

The classification and nomenclature of viruses, as managed by the ICTV, aims to provide a stable and logical framework in which viral diversity can be structured and referenced for a wide range of uses. In recent times, there have been huge advances in genomics that have transformed our knowledge of virus diversity and evolutionary processes. In response, virus taxonomy has had to change and adapt to ensure that its classification and nomenclature serve the needs of, and remain relevant to, its wider constituency. The internet has transformed how virus taxonomy information can be disseminated, and the ICTV website is now the definitive source of current and historical virus taxonomy and serves as a portal for a variety of resources to interrogate the entire ICTV taxonomic database. Virus taxonomy is a dynamic and exciting science. Take a look.

## Abbreviations

EC: Executive Committee
ICTV: International Committee on Taxonomy of Viruses
MSL: Master Species List
SG: Study Group
VMR: Virus Metadata Resource
